# Circulating Vascular Cell Adhesion Molecule-1 (sVCAM-1) Is Associated With Left Atrial Remodeling in Long-Distance Runners

**DOI:** 10.3389/fcvm.2021.737285

**Published:** 2021-11-01

**Authors:** Felipe Contreras-Briceño, Sebastián Herrera, Julian Vega-Adauy, Manuel Salinas, María Paz Ocaranza, Jorge E. Jalil, Jorge Mandiola, Lorena García, Mario Chiong, Pablo F. Castro, Sergio Lavandero, Luigi Gabrielli

**Affiliations:** ^1^Division of Cardiovascular Diseases, Advanced Center for Chronic Diseases (ACCDiS), Faculty of Medicine, Pontificia Universidad Católica de Chile, Santiago, Chile; ^2^Laboratory of Exercise Physiology, Department Health of Science, Faculty of Medicine, Pontificia Universidad Católica de Chile, Santiago, Chile; ^3^Advanced Center for Chronic Diseases (ACCDiS) and CEMC, Faculty of Chemical and Pharmaceutical Sciences & Faculty of Medicine, University of Chile, Santiago, Chile; ^4^Cardiology Division, Department of Internal Medicine, University of Texas Southwestern Medical Center, Dallas, TX, United States

**Keywords:** arrhythmias, cardiac remodeling, exercise, sports cardiology, athletes heart

## Abstract

**Introduction:** An increased risk of atrial fibrillation (AF) has been demonstrated in high-performance athletes. Soluble vascular adhesion molecule-1 (sVCAM-1), a biomarker involved in inflammation and cardiac remodeling, is associated with the development of AF in the general population. However, the relationship between sVCAM-1 and left atrial (LA) remodeling has been poorly investigated in long-distance runners (LDR).

**Aim:** To determine the association between LA remodeling and sVCAM-1 levels in LDR during the training period before a marathon race.

**Methods:** Thirty-six healthy male LDR (37.0 ± 5.3 years; 174.0 ± 7.0 height; BMI: 23.8 ± 2.8; V°O_2_-peak: 56.5 ± 7.3 mL·kg^−1^·min^−1^) were evaluated in this single-blind and cross-sectional study. The LDR were separated into two groups according to previous training levels: high-training (HT) (*n* = 18) ≥100 km·week^−1^ and low-training (LT) (*n* = 18) ≥70 and <100 km·week^−1^. Also, 18 healthy non-active subjects were included as a control group (CTR). In all participants, transthoracic echocardiography was performed. sVCAM-1 blood levels were measured baseline and immediately finished the marathon race in LDR.

**Results:** HT showed increased basal levels of sVCAM-1 (651 ± 350 vs. 440 ± 98 ng·mL^−1^ CTR, *p* = 0.002; and vs. 533 ± 133 ng·mL^−1^ LT; *p* = 0.003) and a post-marathon increase (ΔsVCAM-1) (651 ± 350 to 905 ± 373 ng·mL^−1^; *p* = 0.002), that did not occur in LT (533 ± 133 to 651 ± 138 ng·mL^−1^; *p* = 0.117). In LDR was a moderate correlation between LA volume and sVCAM-1 level (rho = 0.510; *p* = 0.001).

**Conclusions:** In male long-distance runners, sVCAM-1 levels are directly associated with LA remodeling. Also, the training level is associated with basal sVCAM-1 levels and changes after an intense and prolonged exercise (42.2 km). Whether sVCAM-1 levels predict the risk of AF in runners remains to be established.

## Introduction

Moderate aerobic exercise is considered an essential element in maintaining cardiovascular health (1). However, when performed regularly at high intensity, it can have deleterious effects (2). In this regard, there is a growing group of athletes who perform several hours of intense exercise a week and are developing cardiac level changes, a phenomenon called “*athlete's heart”* (3). These structural modifications are characterized by increases in the size and thickness of both ventricular cavities, hypertrabeculation of the left ventricular (LV) walls (4–6), and increases in left atrial (LA) size and changes in its function (6). These physiological adaptations enable the achievement of greater cardiac output during exercise with the consequential increase in the maximum oxygen consumption (V°O_2_-max) and improvement in sports performance (7). However, in some athletes, intense and prolonged exercise can induce unexpected changes in response to physical exercise, the long-term impact of which is not yet clear (8–12). These changes are characterized by exaggerated cardiac remodeling, a phenomenon called “*Phidippides cardiomyopathy”* (3), that it is currently a hypothesis supported by evidence of potentially adverse cardiac remodeling in some endurance athletes (10). In that regard, evidence has shown that intense exercise can generate potentially adverse cardiac remodeling (11), fibrosis of the myocardial tissue (12), and a higher incidence of atrial fibrillation (AF) (13).

Soluble vascular cell adhesion molecule-1 (sVCAM-1), a possible biomarker of the process of fibrosis and cardiac remodeling as a consequence of physical exercise (14), plays a key role in the adhesion of inflammatory molecules and transmigration of leukocytes to the vascular intima (15); it can cause endothelial dysfunction, thus affecting the regulation of cardiac blood flow. In this regard, aerobic exercise has been shown to increase its expression and induce cellular infiltration and inflammation of the heart tissue in untrained subjects (16) as well as patients with peripheral vascular disease (17). On the other hand, both atrial fibrillation (AF) and rapid atrial stimulation are associated with increases in the endocardial expression of VCAM-1 (18); even its elevated plasma levels have been considered a predictor of postoperative AF (19).

The association between sVCAM-1 and cardiac structural changes has not been studied in long-distance runners, who are accustomed to intense and prolonged training. Thus, the present study determined the plasma levels of sVCAM-1 in long-distance runners at different training levels before and immediately after intense and prolonged exercise (42.2 km marathon race) and evaluated the association with LA remodeling on echocardiography. The study aimed to evaluate the role of sVCAM-1 as a possible biomarker of exaggerated atrial remodeling and subsequent development of AF in high-performance athletes.

## Materials and Methods

This prospective single-blind cohort study evaluated 36 Caucasian male competitive long-distance runners. The inclusion criterion was prior participation in at least 3–5 marathon competitions in the last 5 years. The exclusion criteria were as follows: arterial hypertension (resting blood pressure >140/90 mmHg in two separate measurements); dyslipidemia (total cholesterol >200 mg·dL^−1^, low-density lipoprotein cholesterol >100 mg·dL^−1^, high-density lipoprotein cholesterol <40 mg/dL, triglycerides >150 mg·dL^−1^); diabetes mellitus; insulin resistance (homeostatic model assessment >2.5); any degree of smoking; previous cerebrovascular disease (self-reported clinical history); alcohol consumption >40 g per day; drug use; nutritional supplement use; impaired renal function (glomerular filtration rate <60 mL·min^−1^ Modification of Diet in Renal Disease); family history of sudden death; liver damage; autoimmune disease; active neoplasm; chronic obstructive pulmonary disease; diseases that alter the studied biomarker levels (acute inflammation or infectious disease in the month before the marathon); and the use of any antihypertensive, anorectic, antidepressant, and/or antibiotic.

**Figure 1:**
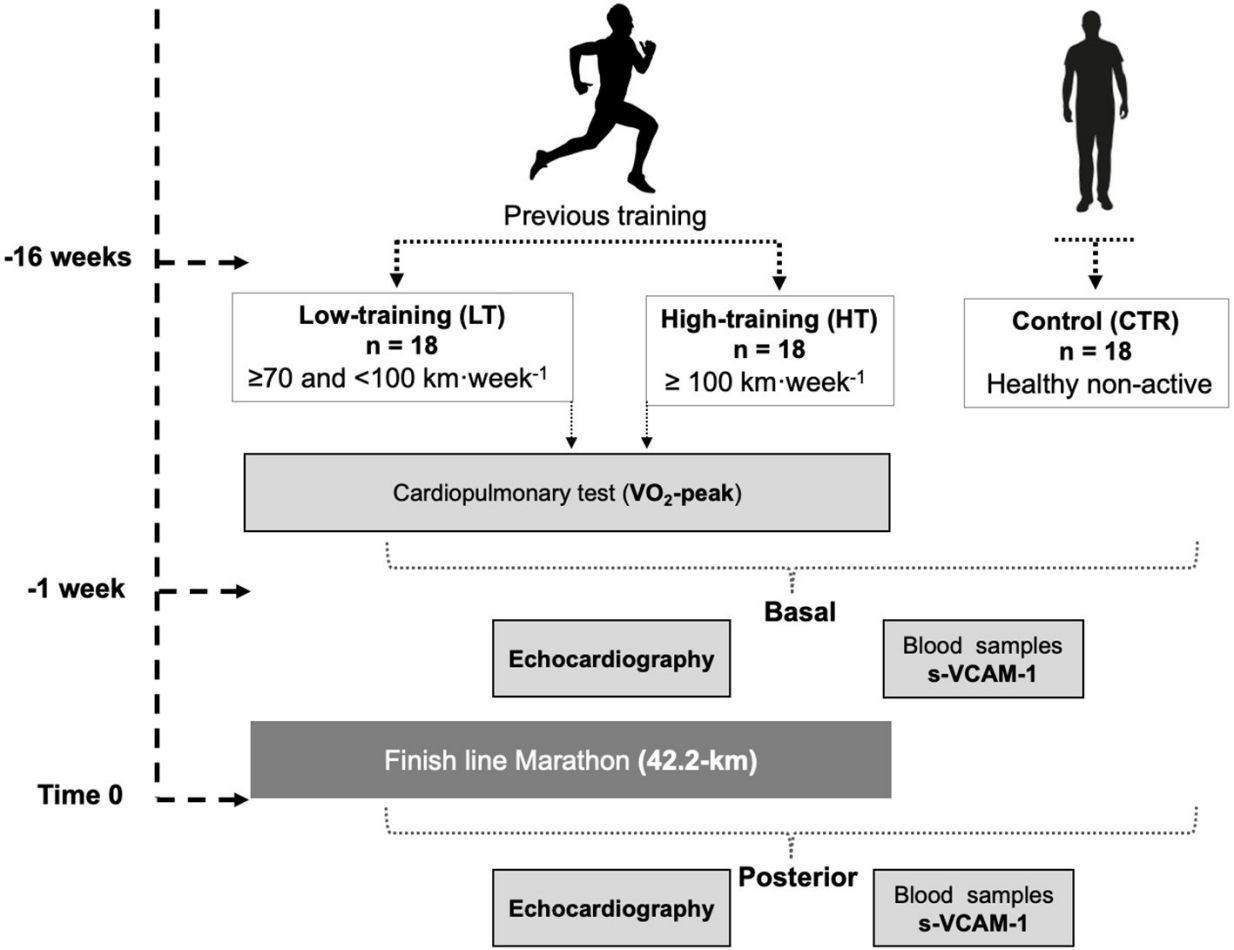
Study scheme design.

The runners were divided into two groups according to previous training levels: high-training (HT; >100 km per week; *n* = 18) and low-training (LT; >70 and <100 km per week; *n* = 18). Eighteen age- and body surface area—matched healthy and non-active men were included as a control group (CTR). This study adhered to the principles outlined in the Declaration of Helsinki and was approved by the Ethics Committee for Human Research at the Faculty of Medicine of the Pontificia Universidad Católica de Chile (project no. 16082603). Written informed consent was obtained from all participants prior to any procedure. The Figure 1 shows the protocol design.

### Cardiopulmonary Test (Peak Oxygen Consumption (V°O_2_-Peak)

To evaluate the performance of runners, the peak aerobic capacity test (V°O_2_-peak) was performed at the end of the “optimal phase” training period. All runners were instructed not to perform physical activity for 24 h prior to the measurement and to avoid intakes of alcohol, caffeine, or other stimulants and food for at least 3 h before. The V°O_2_-peak test was measured on a treadmill ergometer (HP Cosmos, Traunstein, Germany) until voluntary exhaustion, despite oral breathing (respiratory quotient, 1.20 ± 0.05). The exercise protocol consisted of a 3-min rest, 5-min warm-up (8 km·h^−1^), and subsequent increase of 2 km·h^−1^ every 150 s, until all criteria for stopping the test were met. The exhaled gases were measured continuously using a metabolic detector (Masterscreen-CPX, Jaeger, Traunstein, Germany) equipped with O_2_ and CO_2_ analyzers.

### Echocardiographic Assessment

Standard transthoracic echocardiography (TTE) was performed in all subjects according to international standards of the American Society for Echocardiography (ASE) (20). The baseline TTE was obtained the week before the marathon. The examination was performed by trained expert personnel and included parasternal and apical views on a 1.5/3.5 MHz cardiac transducer and a Vivid I portable computer (GE, Healthcare, Horton, Norway). Parietal wall thickness, cavitary volumes, and LV mass were evaluated in accordance with ASE recommendations (20). The LV mass was calculated using the linear method (21). To LA volume assessment, the biplane (two and four chamber view) disk summation technique were used, and for RA volume, the single plane disk summation technique in apical four chamber view was used (21). Image quality was optimized for at least 60 frames per second and digitally stored for further analysis using EchoPAC BT 12 software (GE Healthcare, Horton, Norway).

### Assessment of sVCAM-1

A venous blood sample was taken from all subjects. A commercially available immunoenzymatic assay (ELISA sVCAM-1, R&D Systems, Minneapolis, MN, USA) was used to determine the sVCAM-1 plasma levels. Each sample was analyzed twice, and the mean value was obtained using the standard curve method. Blood cell count, liver function, renal function, and plasma electrolytes were also measured using standard methods.

### Statistical Analysis

The data distribution was assessed using the Shapiro-Wilk test. Variables were evaluated using the Mann-Whitney U-test and Kruskal-Wallis test. The associations between variables were evaluated using the Spearman correlation test. To evaluate independent association between LA remodeling with sVCAM-1 levels and training degree parameters, a binary logistic regression was performed; the dependent (categorical) variable for regression was defined as: LA volume ≤ 41 mL·m^−2^ or LA volume >41 mL·m^−2^ (the cut-off of moderate LA dilatation) (21). Values of *p* < 0.05 were considered significant. The statistical software used was GraphPad PRISM v.8.0 (GraphPad Software Inc., San Diego, CA, USA).

## Results

The participants' characteristics are shown in Table 1. All athletes ended the race without symptoms or signs of adverse events. The groups were similar in age, and no differences were noted in the blood and biochemical parameters. The CTR group subjects had a higher mean heart rate than the athletes (*p* < 0.001 vs. HT and LT). The mean V°O_2_-peak was higher in the HT group (*p* = 0.020 vs. LT).

**Table 1:** Characteristics of the participants.

	**Groups**			***p*-value**
	**CTR**	**HT**	**LT**	
Age (years)	36 ± 4	37 ± 6	38 ± 5	0.373
Height (cm)	175 ± 6	174 ± 6	172 ± 7	0.470
Weight (kg)	72 ± 9	71 ± 8	69 ± 8	0.090
Body superficial area (m^2^)	1.9 ± 0.1	1.8 ± 0.1	1.8 ± 0.1	0.075
Heart rate at rest (bpm)	69 ± 6^*^	53 ± 8	55 ± 7	<***0.001***
Na^+^ (mEq·L^−1^)	142 ± 2	142 ± 3	142 ± 2	0.440
Creatinine (mg·dL^−1^)	0.99 ± 0.11	0.98 ± 0.09	0.97 ± 0.10	0.630
Aspartate aminotransferase (U·L^−1^)	26 ± 7	29 ± 9	28 ± 8	0.670
Hematocrit (%)	42 ± 3	43 ± 2	43 ± 3	0.870
Uric acid (mg·dL^−1^)	5.2 ± 0.8	5.6 ± 0.9	5.0 ± 0.9	0.170
V°O_2_-peak (ml·kg^−1^·min^−1^)	−−	58.5 ± 5.3^#^	52.5 ± 8.1	***0.020***
Running experience (years)	−−	17 ± 3	16 ± 3	0.810
Time training per week (hours)	−−	19 ± 2^#^	15 ± 2	***0.018***
Training intensity (%HR máx., 220-age)	−−	81 ± 3	80 ± 2	0.780

*Data are expressed as mean and standard deviation*.

* *P < 0.05 control vs. other groups; Kruskal-Wallis test*.

# *P < 0.05 HT vs. LT; Mann-Whitney U-test*.

*CTR, Control; HT, High-training group (≥100 km by week); LT, Low-training group (≥70 and <100 km by week); V°O_2_-peak, Peak oxygen consumption*.

*The meaning of numbers in bold and italic are related to difference statistically (p-value less than 0.05)*.

### Exercise and Cardiac Remodeling

Significant structural changes were observed in the HT group than in the LT and CTR groups, specifically greater interventricular septum (*p* < 0.001) and posterior wall (*p* < 0.005) thickness and a higher LV mass index (*p* < 0.001) and LA volume index (*p* < 0.001). Also, RA volume index was higher in HT group as compared to CTR group (*p* = 0.030) (Table 2).

**Table 2:** Echocardiographic characteristics of participants.

**Heart chambers quantification**	**Groups**			***p*-value**
	**CTR**	**HT**	**LT**	
Diastolic diameter LV (mm)	46 ± 4	49 ± 5	48 ± 5	0.404
Systolic diameter LV (mm)	30 ± 4	29 ± 5	30 ± 5	0.879
Posterior wall (mm)	8.1 ± 0.8	9.1 ± 1.2^*^	8.2 ± 1.1	<***0.005***
Interventricular septum (mm)	7.6 ± 0.8	9.3 ± 2.1^*^	8.5 ± 1.2	<***0.001***
Ejection fraction (%)	57 ± 4	55 ± 3	55 ± 6	0.110
LV mass index (gr·m^−2^)	58 ± 11	106 ± 27^*^	78 ± 18	<***0.001***
LA volume index (mL·m^−2^)	25 ± 9	42 ± 8^*^	30 ± 11	<***0.001***
LA diameter (mm)	33 ± 4	36 ± 4	34 ± 3	0.220
E wave (cm·s^−1^)	77 ± 15	78 ± 13	84 ± 12	0.217
A wave (cm·s^−1^)	48 ± 16	50 ± 12	53 ± 10	0.438
Deceleration time (ms)	221 ± 66	233 ± 65	229 ± 65	0.184
RA volume index (mL·m^−2^)	21 ± 9	43 ± 11^#^	33 ± 10	***0.030***

*Data are expressed as mean and standard deviation*.

* *P < 0.05 HT vs. other groups*.

# *P < 0.05 HT vs. CTR. Kruskal-Wallis test*.

*CTR, Control; HT, High-training group (≥100 km by week); LT, Low-training group (≥70 and <100 km by week); LV, left ventricular; LA, left atrial; RA, right atrial*.

*The meaning of numbers in bold and italic are related to difference statistically (p-value less than 0.05)*.

### Pre- and Post-marathon sVCAM-1 Plasma Levels

**Figure 2:**
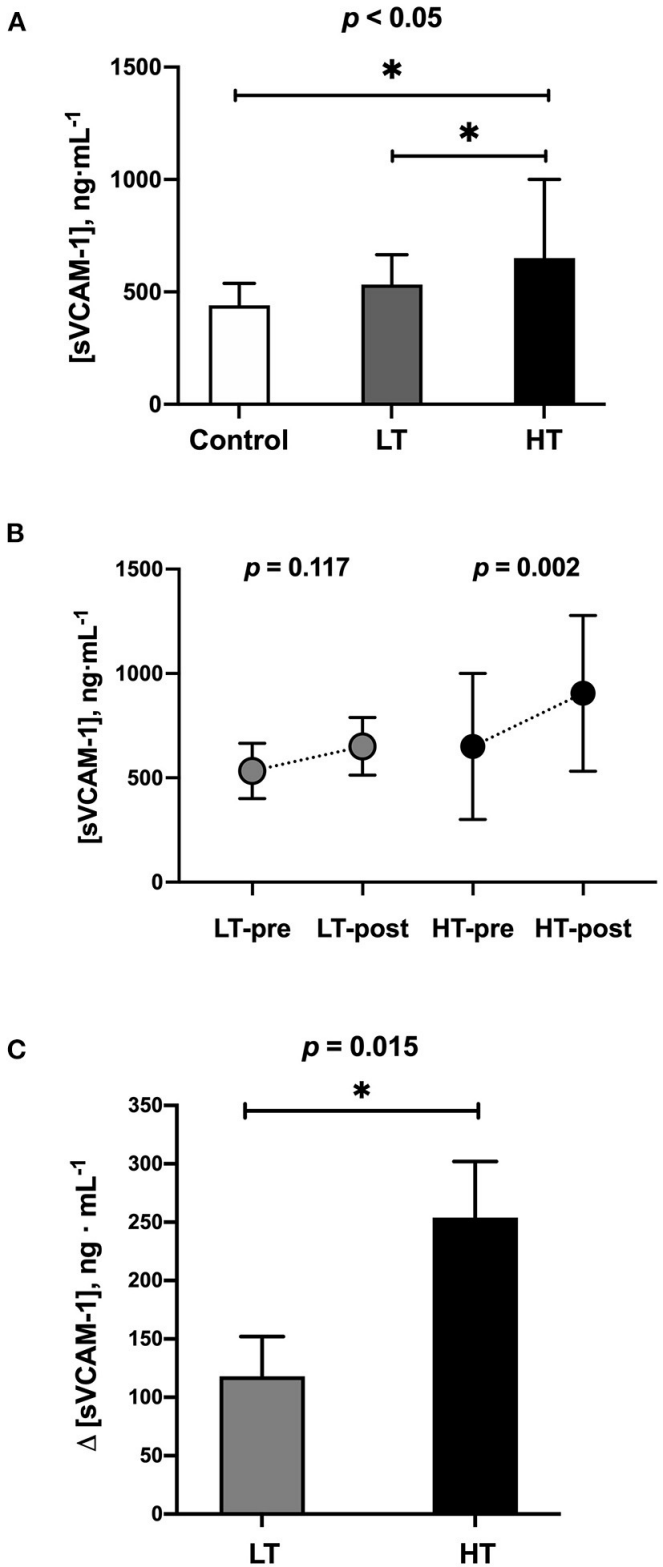
Blood samples levels of sVCAM-1. **(A)** At baseline conditions (1 week before marathon race). **(B)** Exercise-induced changes after marathon race (42.2 km). **(C)** Comparison of changes (ΔsVCAM-1 = post–pre-marathon) between runners' groups. LT, low training (≥70 and <100 km·week^−1^) (*n* = 18); HT, high training (≥100 km·week^−1^) (*n* = 18). * = *p*-value < 0.05.

The mean resting sVCAM-1 plasma levels were higher in the HT vs. LT and CTR groups (651 ± 350 vs. 566 ± 133 and 440 ± 98 ng·mL^−1^, respectively; *p* < 0.05). After the exercise was completed, an increase in the HT group was observed (from 651 ± 350 to 905 ± 373 ng·mL^−1^, *p* = 0.002). The LT group showed a similar trend, although non-significant. The comparison between HT vs. LT groups of changes in sVCAM-1 plasma levels (ΔsVCAM-1 = post–pre-marathon) was significant (254 ± 48 vs. 118 ± 34, respectively; *p* = 0.015) (Figure 2).

### Multivariate Analysis for LA Remodeling in Athletes

In the multivariate analysis for LA volume (as a categorical variable previously described), the following variables were assessed: sVCAM-1 at baseline, age, running experience (years), time training per week (hours), training intensity (%HR máx. 220-age) and V°O_2_-peak. In the final model the significant variables were: sVCAM-1 (OR: 2.85; *p* < 0.010) and V°O_2_-peak (OR: 1.75; *p* = 0.02).

### Correlation Between Atrial Size and Baseline sVCAM-1 Levels

**Figure 3:**
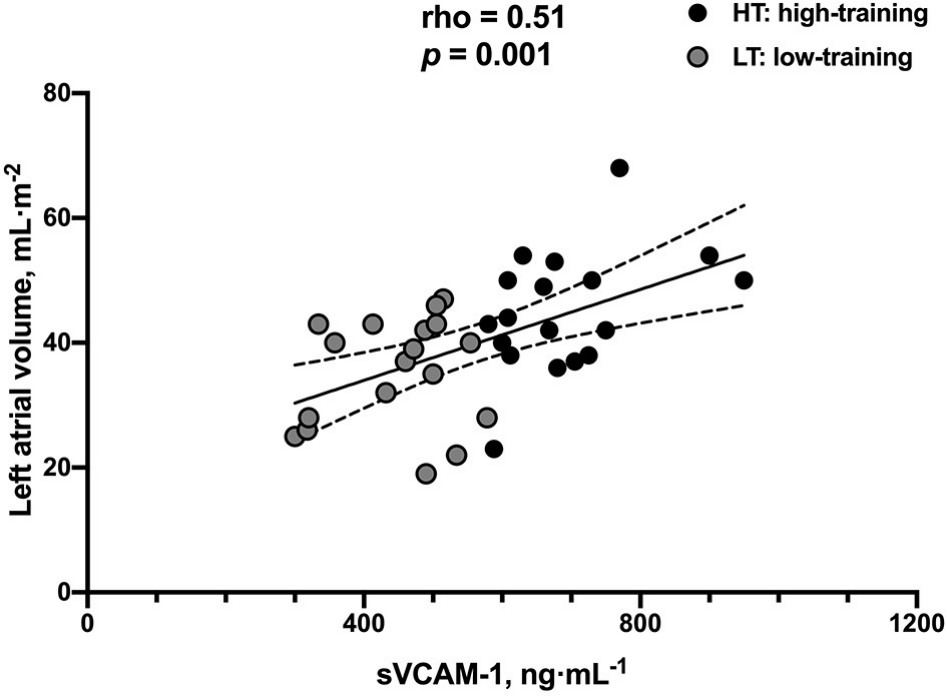
Correlation between sVCAM-1 blood sample levels and left atrial volume in long-distance runners (*n* = 36).

In long-distance runners, there was a moderate correlation between LA volume and baseline plasma sVCAM-1 levels (rho = 0.510, *p* = 0.001) (Figure 3), showing that the LA remodeling process is related at least in part to sVCAM-1 levels. No significant correlation between RA volume and sVCAM-1 was found.

## Discussion

The main finding of this study is that runners in the HT group had elevated sVCAM-1 plasma values compared to runners in the LT group and the healthy physically non-active subjects (CTR group). In addition, in both runner groups, there was an increase in sVCAM-1 after intense and prolonged exercise (42.2 km marathon), most significantly in the HT group. The sVCAM-1 plasma values were directly correlated with the LA volume evaluated by echocardiography.

Moderate exercise is considered a key element in maintaining cardiovascular health (1) and an important tool in cardiovascular rehabilitation programs for patients with coronary heart disease (22) and chronic heart failure (23). International guidelines suggest 150 min per week of moderate exercise or 75 min per week of vigorous exercise for the general adult population (24). However, a growing group of people performs 20 or more hours of intense exercise per week. These people demonstrate multiple adaptive cardiac changes, a condition called “athlete's heart” (25, 26). This cardiac remodeling process is an adaptation to the volume overload inherent to aerobic physical training; in most cases, it is considered a reversible and benign condition (27).

In this study, we found that only HT athletes, not LT athletes, have significant changes in LV cardiac remodeling and LA volume. This result is concordant with previous data showing that intense training induces significant changes in the size and function of the atrial syncytium (6). Pellicia et al. showed 20% mild atrial dilation and 2% severe LA dilation in a group of 1,777 competitive athletes (25). Our group included professional handball players (26) and marathon runners (9, 28) with significant right atrial dilation; however, in this study, the parameters of atrial deformation were preserved, unlike other pathological conditions with similar atrial dilation (29, 30). On the other hand, our results show that although the most trained runners had a higher-volume LA and thicker ventricular walls, they had a normal ejection fraction similar to the other study participants.

This study did not specifically evaluate LA function, which constitutes a limitation; however, we previously described that this function is particularly stressed during the performance of aerobic resistance exercise in trained athletes with characteristics similar to the runners studied here (8), a finding that is directly associated with V°O_2_-peak and sports performance (31). Our findings suggest that athletes with high-performance aerobic resistance develop right atrial and LA dilation, a condition that is associated with less atrial deformation during contraction at rest (31). Our research group previously reported that this condition enables greater functional reserve but causes greater atrial wall stress (26). We also describe that a subgroup of athletes showed severe atrial dilation associated with a lower capacity to increase atrial deformation during exercise contraction, possibly resulting in early atrial dysfunction (8).

Although risk prediction models for AF that incorporate clinical and genetic factors have been developed for the general population, their discriminatory ability remains moderate (29, 30). To our knowledge, there are no prediction models in high-performance athletes that stratify the risk of AF; only a few articles report some key factors to consider, such as training history (32), body height, and atrial size (33). Thus, new research is necessary to clarify which characteristics are associated with an increased risk of AF in highly trained athletes, and the measurement of biomarkers associated with AF in athletes seems to be an interesting study topic.

In different clinical scenarios, LA dilation and deformation properties are related to the risk of AF (32–36), a phenomenon that could be extrapolated to the sports setting. In addition to LA dilation, other potentially arrhythmogenic changes can occur in the athlete's atrium, including fibrosis that promotes conduction heterogeneity (36), changes in autonomic equilibrium toward parasympathetic activation and a decrease in sympathetic tone shortening of the atrial refractory period (37), and activation of ectopic foci facilitated by autonomous changes (38). These electrical and structural changes within the atrium facilitate the re-entry mechanism and AF that still require confirmation with new studies in high-performance athletes (38). However, it is challenging to identify the athletes with the highest risk of developing the arrhythmia because the absolute risk of AF among them is relatively low for any subject (3% among endurance athletes) (39). Risk prediction models for AF in the general population have been developed, however they show only a moderate discriminatory capacity (29, 30). Currently there are no prediction models to stratify the risk of AF in endurance athletes, with only few reports considering individual life time training history (32), atrial dilatation and body stature (33), but without a clear threshold beyond which the risk of AF increases.

From a clinical point of view, sVCAM-1 is a biomarker significantly associated with the risk of AF in the general population (40, 41) as well as postoperative AF (22, 42). In fact, sVCAM-1 was the only biomarker that was significantly associated with long-term risk of AF, independent of a large number of clinical and laboratory measurements (43). Willeit et al. in a population-based cohort study with a 20-year follow-up described that sVCAM-1, but not other inflammation markers, are significantly associated with new-onset AF in the general community (40).

Our results show that the more trained runners showed higher baseline sVCAM-1 levels than the moderately trained and physically active healthy subjects (Figure 2A). Although VCAM-1 levels are low in the endothelium and the resting endocardium, its expression may increase under multiple stress conditions, including high-intensity aerobic exercise (14). The increase of this biomarker leads to cell infiltration and inflammation of the entire heart, stimulating the process of fibrosis and cardiac remodeling. This has also been seen in experimental models in which both AF and rapid atrial stimulation increase the endocardial expression of VCAM-1 (18). Accordingly, increased ventricular tissue VCAM-1 have been associated with cardiac remodeling due to hemodynamic stress (44).

Therefore, an association among strenuous exercise, cardiac remodeling, sVCAM-1 and AF is proposed. However, we do not know whether elevated sVCAM-1 values at rest in more trained runners are predictors of future AF. For that purpose, it will be necessary to perform new studies with clinical follow-up in this population.

Another important finding was that highly trained runners significantly increased sVCAM-1 levels at the end of the marathon (Figure 2B), which was higher than low trained runners (Figure 2C). A previous investigation reported that after brisk exercises (similar to high-intensity interval exercises), the levels of VCAMs increase in trained subjects (45), returning to basal levels promptly (48-h post-exercise); and recently, an interesting review reported that the low-to-moderate intensity aerobic exercise favorably decreases VCAMs levels in a variety of subject populations, while VCAMs levels momentarily increase immediately following high-intensity aerobic exercise, returning pre-exercise levels within several hours post-exercise. On the other hand, regardless of its intensity, resistance exercise does not significantly change the VCAMs levels (46). To our knowledge, there are no previous studies in runners chronically accustomed to that type of stimulus that has evaluated the changes in sVCAM-1 levels after an intense and prolonged exercise, such as a marathon race. In the clinical context, there has been a ventricular level increase of VCAM-1 associated with hemodynamic stress in patients with rheumatoid arthritis (44) or intermittent claudication (47). In skeletal muscle tissue, an increase has been seen in post-prolonged exercise (48) associated with muscle remodeling (49).

Another interesting finding is the moderate correlation found between LA volume and sVCAM-1 levels in this group of athletes (Figure 3) since it can be inferred that VCAM-1 blood levels may have a role as a biomarker for LA remodeling link to AF risk in aerobic resistance athletes, which should be explored in future studies involving both a greater number of participants and both sexes. Also, the evaluation of LA function (contractile, conduit, and reservoir) and its relationship with sVCAM-1 could contribute to a better characterization of these athletes. Considering that multiple factors linked to the degree of training, like duration, volume, the intensity of exercises, and oxygen consumption, could be implicated in the changes induced by exercise in cardiac chambers in our participants; besides the simple associations showed, we did a multivariate analysis to find associations between these variables and sVCAM-1 levels and LA remodeling. This analysis showed that sVCAM-1 levels and V°O_2_-peak were independent predictors of LA volume; this finding corroborates that LA remodeling in athletes is a complex phenomenon probably related to the degree of training and individual predisposition but at least in part linked to sVCAM-1 levels. Regarding the right atrial volume, we found a higher volume in the more trained subjects compared to the controls, but with a greater dispersion, these findings being consistent with previous studies (9–11).

In conclusion, high-intensity physical exercise is associated with an increase in sVCAM-1 plasma levels in Caucasian male runners. Because sVCAM-1 levels were positively correlated with higher LA volume, sVCAM-1 could be a potential biomarker that could be used to assess potential adverse consequences for LA structure and function in high-performance athletes. In addition, since sVCAM-1 has been established as a predictor of postoperative AF and AF in the general population, we propose that this biomarker could be used to predict the risk of AF in athletes, which requires further prospective studies to validate our findings.

## Data Availability Statement

The raw data supporting the conclusions of this article will be made available by the authors, without undue reservation.

## Ethics Statement

The studies involving human participants were reviewed and approved by Ethics Committee of Pontificia Universidad Católica de Chile (Chile) (project no. 16082603). The patients/participants provided their written informed consent to participate in this study.

## Author Contributions

FC-B, LG, JV-A, MC, PC, and SL substantially contributed to the concept and design of the study. MO and LG contributed to assay setup. JV-A, SH, and MS contributed to data acquisition, analysis, and interpretation. FC-B, LG, MO, MC, PC, JJ, and SL contributed to the data interpretation, discussion, and manuscript preparation. FC-B, LG, JJ, PC, and SL wrote the manuscript. All authors critically revised the manuscript for important intellectual content and contributed to the article and approved the submitted version.

## Funding

This work was supported by grants from the Fondo Nacional de Ciencia y Tecnología (FONDECYT 1170963 to LG, Anillo ACT192144 to MO, FONDAP 15130011 to PC, LG, MC, MO, and SL from the Agencia Nacional de Investigación y Desarrollo [ANID], Chile) and funds for translating and editing from the Pontificia Universidad Católica de Chile (VRI) to FC-B.

## Conflict of Interest

The authors declare that the research was conducted in the absence of any commercial or financial relationships that could be construed as a potential conflict of interest.

## Publisher's Note

All claims expressed in this article are solely those of the authors and do not necessarily represent those of their affiliated organizations, or those of the publisher, the editors and the reviewers. Any product that may be evaluated in this article, or claim that may be made by its manufacturer, is not guaranteed or endorsed by the publisher.
